# Hemodynamic Stability, Patient Acceptance and Cost of Intravenous Propofol and Inhalational Sevoflurane for Induction of Anaesthesia: A Prospective, Randomized Comparative Study

**DOI:** 10.7759/cureus.7687

**Published:** 2020-04-16

**Authors:** Kirtibala Dhande, Jitendra Kshirsagar, Ashish Dhande, Narendra Patil, Parvati V

**Affiliations:** 1 Anesthesiology, DY Patil Hospital, Navi Mumbai, IND; 2 Anesthesiology, Deenanath Mangeshkar Hospital, Pune, IND; 3 Urology, DY Patil University - School of Medicine, Navi Mumbai, IND; 4 Anesthesiology, DY Patil University - School of Medicine, Navi Mumbai, IND

**Keywords:** sevoflurane, propofol, induction, hypotension, acceptance, pharmacoeconomics, hemodynamic stability

## Abstract

Introduction

The effects of an anesthetic agent on the hemodynamic stability are of prime importance in patients with compromised hemodynamics. Although comparative studies of sevoflurane and propofol are reported, most of these are aimed to assess maintenance and early postoperative recovery. There are very few studies on hemodynamic changes occurring with these two agents. This study compares the hemodynamic stability, patient acceptance, and cost of intravenous (IV) propofol versus inhalational (IH) sevoflurane for the induction of anesthesia.

Methods

This prospective, randomized comparative study was conducted among 80 patients with American Society of Anaesthesiologists (ASA) grade-I requiring general anesthesia (GA) for elective surgical procedures. The study was approved by the institutional ethics committee and was conducted as per the principles of the Declaration of Helsinki and Good Clinical Practice (GCP) guidelines. Enrolled patients were randomized to receive either intravenous (IV) propofol 2 mg/kg (n=40) or gradual inhalational (IH) induction with sevoflurane (n=40). All patients were maintained with sevoflurane 2% in 67% nitrous oxide (N_2_O) and O_2_. Hemodynamic parameters like pulse rate and mean arterial pressure (MAP) were monitored every minute up to five minutes. Patients' acceptance was assessed on a 10-item questionnaire, and the cost of anesthesia was assessed based on the anesthetic requirement. The hemodynamic parameters were compared between the two groups using two-way repeat-measures ANOVA. The incidence of hypotension was compared using Fischer’s test.

Results

The two groups were similar at baseline with respect to the demography and other baseline characteristics. There was greater (p<0.05) fall in MAP with propofol induction (28.48%) compared to sevoflurane (14.61%). Greater reduction in pulse rate (p<0.05) with sevoflurane (9.18) induction was observed compared to propofol (5.28). Patient acceptance for both drugs was similar (p>0.05). Although sevoflurane was unpleasant, propofol injection was painful. Ninety percent of patients preferred propofol for repeat anesthesia as against 85% of patients with sevoflurane. Considering the quantity of anesthetic consumed and the unit cost, propofol was more costly as compared to sevoflurane.

Conclusion

Sevoflurane maintains better hemodynamic stability compared to propofol, and patient acceptance of both drugs is similar. Induction with sevoflurane was found to be cheaper as compared to propofol induction.

## Introduction

Induction of anesthesia is an important activity in a surgical event, and there are high chances of hemodynamic instability, hypoxia, arrhythmias, and excitatory reflexes. Thus, the induction process should be quick and devoid of any such effects, especially in compromised patients. Although an ideal inducing agent is yet to be seen, the ideal requirements change based on the surgery, pathophysiological condition of the patient, and availability of equipment. An anesthesiologist must wisely select from the available drugs, one that best suits the particular patient.

Intravenous propofol has been the drug of choice for the induction of anesthesia due to its safety profile, relaxation, depression of upper airway reflexes, and mild bronchodilation [1]. However, it may cause adverse effects such as cardiovascular depression leading to hemodynamic instability, pain on injection, thrombophlebitis, and respiratory depression [2]. Sevoflurane is a halogenated volatile anesthetic agent which is non-irritating to the respiratory tract, has the highest hemodynamic stability, and has bronchodilator activity [3]. Sevoflurane is the best volatile inducing agents with faster induction and rapid recovery [4]. Thus, both propofol and sevoflurane have their own merits and limitations.

Although many investigators have done a comparison of sevoflurane and propofol for their effects on hemodynamic stability, there are no studies which report acceptance of these inducing agents by patients and the cost of inducing agents. We conducted this prospective, randomized study to compare the hemodynamic stability, patient acceptance, and cost of intravenous (IV) propofol versus inhalational (IH) sevoflurane for induction of anesthesia.

## Materials and methods

Study design and setting

This randomized, comparative study was carried out after obtaining approval from the institutional ethics committee (IEC). The study was conducted following the principles of the Declaration of Helsinki (World Medical Association) and Good Clinical Practice (GCP) guidelines issued by the Indian Council of Medical Research (ICMR) and the Drugs Controller General of India (DCGI). The study procedures were explained to all patients and informed consent was obtained.

Study subjects

Patients of either gender, between 20 to 60 years of age, patients of American Society of Anaesthesiologists (ASA) grade I posted for elective surgery and requiring general anesthesia were enrolled after obtaining written informed consent. Patients with allergy to either propofol or sevoflurane or any other drugs were excluded. A total of 80 study eligible patients were randomized to receive IV propofol (n=40) or inhalational sevoflurane (n=40) for induction of anesthesia. The sample size was not based on any estimations and assumptions, and it was planned to perform posthoc power analysis for the primary outcome of the study. 

Randomization and blinding

Block randomization (block of 40 with two blocks) was done using PC based (Rando V1.0 for MS Windows) predetermined randomization schedule. The randomization was prepared by independent personnel and was concealed in sealed separate envelopes for each study participant. The study team was blinded for the randomization, and allocation was done only after the eligible participant was enrolled and assigned a study serial number. After enrolment, the sealed envelope was opened by the study team members to reveal the treatment allocation for the participant. The study was initiated after randomization was done.

Study procedures

All patients underwent standard multiparameter monitoring for a pulse, blood pressure, respiration, oxygen saturation (SpO_2_), and electrocardiogram (ECG). All patients were premedicated with IV midazolam 0.02 mg/kg, and fentanyl 1.5 mcg/kg [5]. Patients breathed oxygen for one minute through a clear plastic face mask at a flow rate of 5 L/min via co-axial Bain circuit as pre-oxygenation. Propofol group patients received IV propofol at a constant rate of 8-10 ml/min till induction. Sevoflurane group patients were explained about mask induction, and sevoflurane was administered through a transparent face mask starting at 0.5% and incrementally increased by 0.5% every 15 sec in 100% O_2_ at a total gas flow of 5 L/min via co-axial Bain circuit [6]. Induction was confirmed by loss of eyelash reflex. All patients received succinylcholine 2 mg/kg IV prior to tracheal intubation [7]. All intubations were done by the same investigator. All patients in both groups were maintained with 2% sevoflurane and 60% N_2_O in O_2_ at 5 L/minute [8]. An independent observer who was blinded for the treatment recorded the heart rate (HR), systolic blood pressure (SBP), diastolic blood pressure (DBP) at baseline, before midazolam premedication, three minutes after midazolam, pre-induction, post-induction, after intubation and every minute for five minutes after intubation.

Study outcomes

The primary outcome was the hemodynamic stability assessed by changes in mean arterial pressure (MAP), SBP, DBP, and HR. Secondary outcomes were patient acceptance and the cost of induction. Patient acceptance was recorded on a three-point Likert scale - pleasant, indifferent and unpleasant. Patients were asked whether they would prefer the same anesthetic if required in the future. The cost of induction of anesthesia was calculated based on the market unit price, and the total consumption of anesthetic agents.

Statistical analysis

The hemodynamic parameters (HR, SBP, DBP, and MAP) were compared between the two groups using the general linear model (GLM) procedure (repeat measures analysis of covariance - ANCOVA) with inducing agents as independent variable and age, body weight and gender as covariates. The time of measurements was the baseline, before midazolam, three minutes after midazolam, pre-induction, post-induction, after intubation and every minute for five minutes after intubation. Binary data was compared between two groups using Fischer's chi-square test. All analyses were done using two-sided tests at alpha 0.05 (95% confidence level), and the cut-off p-value was 0.05 for tests of significance. Statistical analyses were performed using the Statistical Package for Social Sciences, version 17.0 (SPSS Inc, Chicago, Illinois). 

## Results

The demographic characteristics of patients in the two groups are shown in Table 1. Both groups were similar with respect to the demography and baseline data (p>0.05).

**Table 1:** Demography and baseline data IV - intravenous; IH - inhalational

	IV propofol (n=40)		IH sevoflurane (n=40)		ANOVA	
	Mean	SD	Mean	SD	F	p
Age (years)	33.20	10.83	34.55	9.84	0.341	0.561
Body weight (kg)	59.30	12.08	60.75	10.37	0.332	0.566
Heart rate (per min)	77.63	12.88	76.10	15.39	0.231	0.632
Systolic blood pressure (mm Hg)	126.43	14.70	131.73	12.88	2.941	0.090
Diastolic blood pressure (mm Hg)	78.98	10.74	75.90	10.40	1.692	0.197
Mean arterial pressure (mm Hg)	94.79	11.46	94.51	8.98	0.015	0.902
Gender	No.	%	No.	%	Chi-square	p
Male	12	30.0%	19	47.5%	2.581	0.168
Female	28	70.0%	21	52.5%		

**Figure 1:**
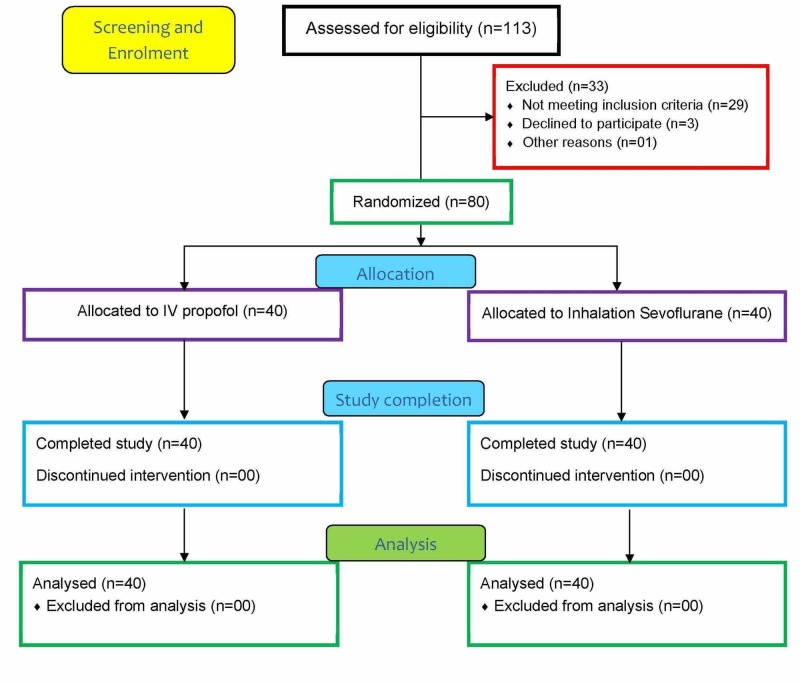
CONSORT diagram CONSORT - Consolidated Standards of Reporting Trials

There we no data loss and all patients (n=80) completed the study as per protocol and were included for analysis Figure 1 (Consolidated Standards of Reporting Trials [CONSORT] diagram).

**Figure 2:**
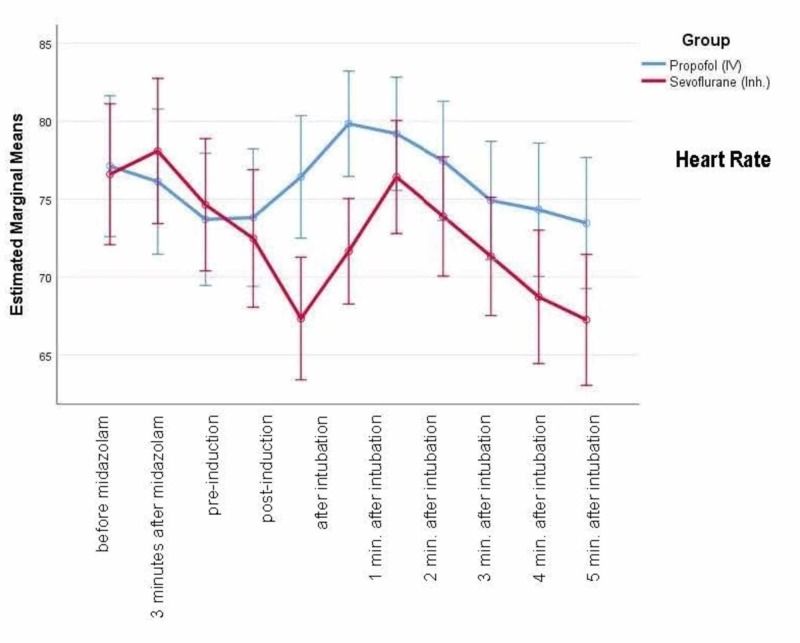
Heart rate at baseline and different time-points

Figure 2 shows the mean heart rate (HR) at baseline and different time-points. There is a reduction in HR with sevoflurane, whereas an increase in HR with propofol after induction - this trend continues up to five minutes after intubation.

**Figure 3:**
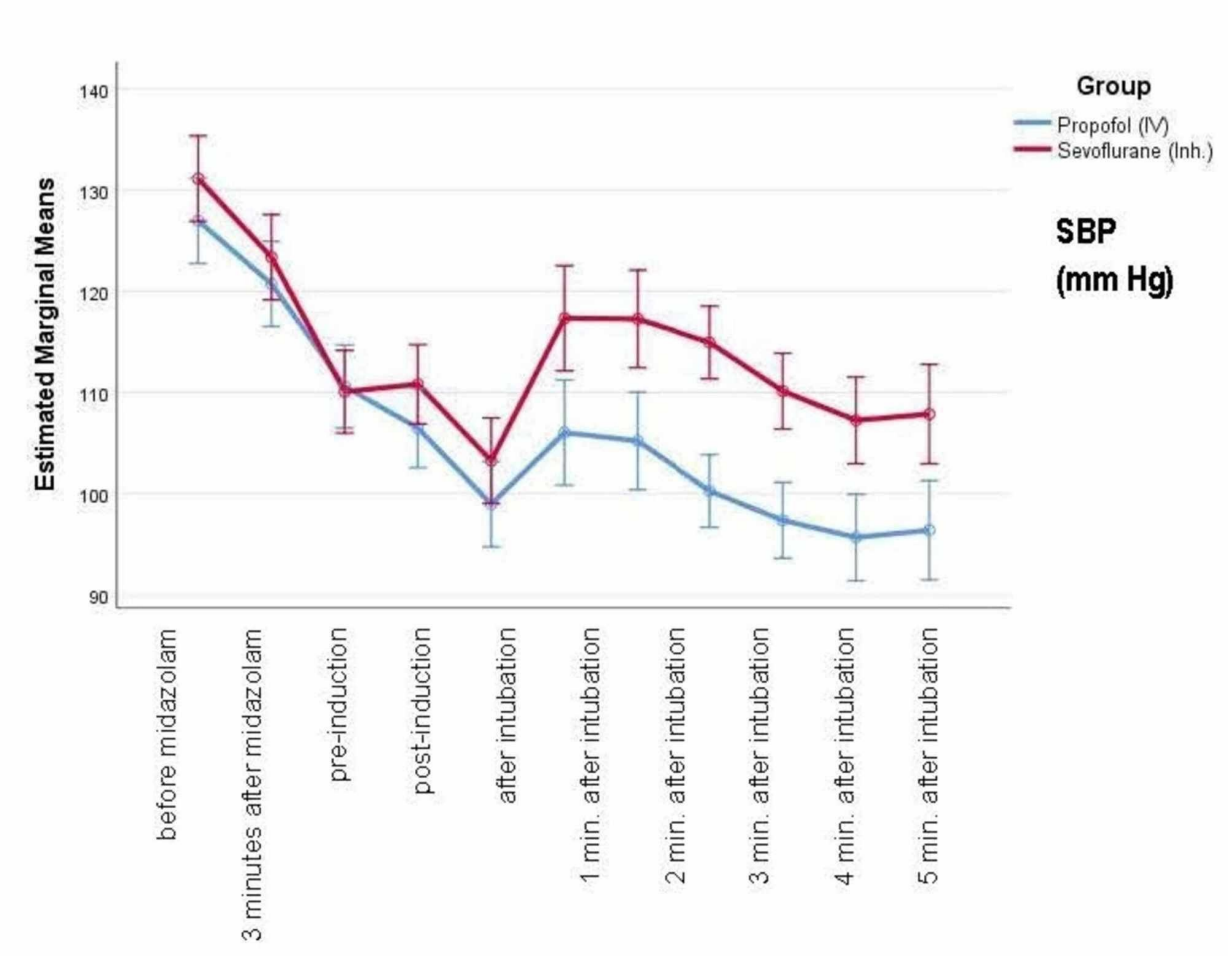
Mean SBP at baseline and different time-points SBP - systolic blood pressure

Figure 3 shows the mean systolic blood pressure (SBP) at baseline and different time-points. There is a greater reduction in SBP with propofol compared to sevoflurane, and this reduction starts immediately after induction and continues to be low up to five minutes after intubation.

**Figure 4:**
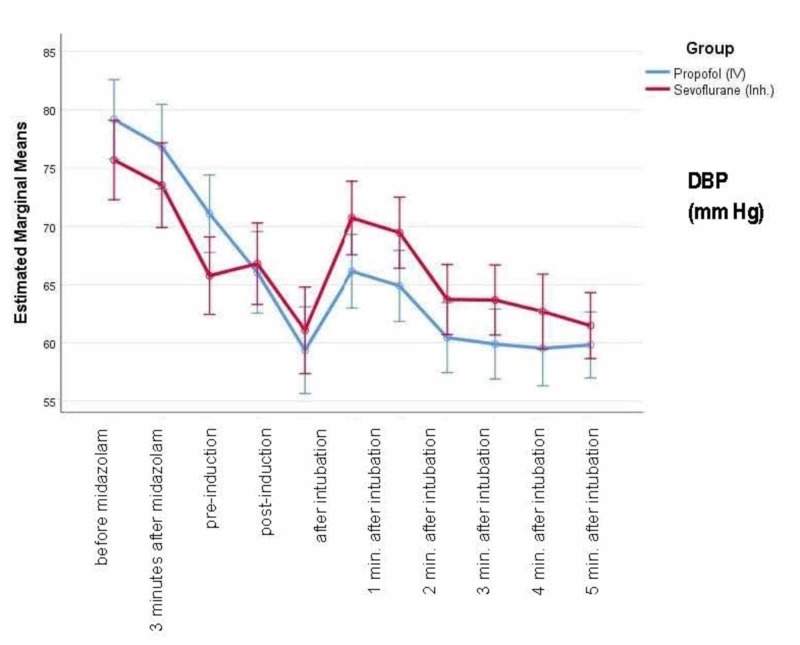
Mean DBP at baseline and different time-points DBP - diastolic blood pressure

Figure 4 shows the mean diastolic blood pressure (DBP) at baseline and different time-points. There is a greater reduction in DBP with propofol compared to sevoflurane, and this reduction starts immediately after induction and continues to be low up to five minutes after intubation.

**Figure 5:**
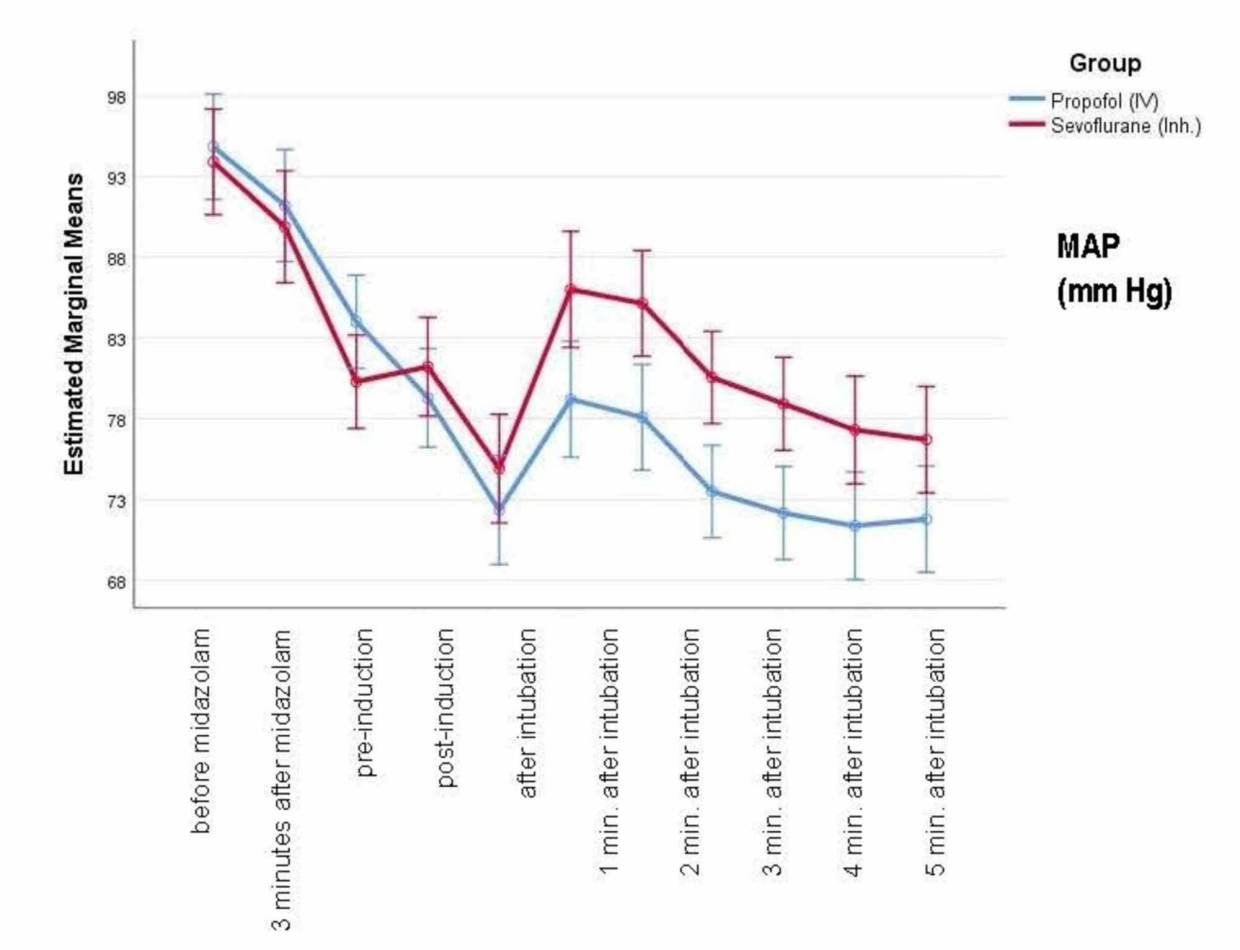
Mean MAP at baseline and different time-points MAP - mean arterial pressure

Figure 5 shows the mean arterial pressure (MAP) at baseline and different time-points. There is a greater reduction in MAP with propofol compared to sevoflurane, and this reduction starts immediately after induction and continues to be low up to five minutes after intubation.

**Figure 6:**
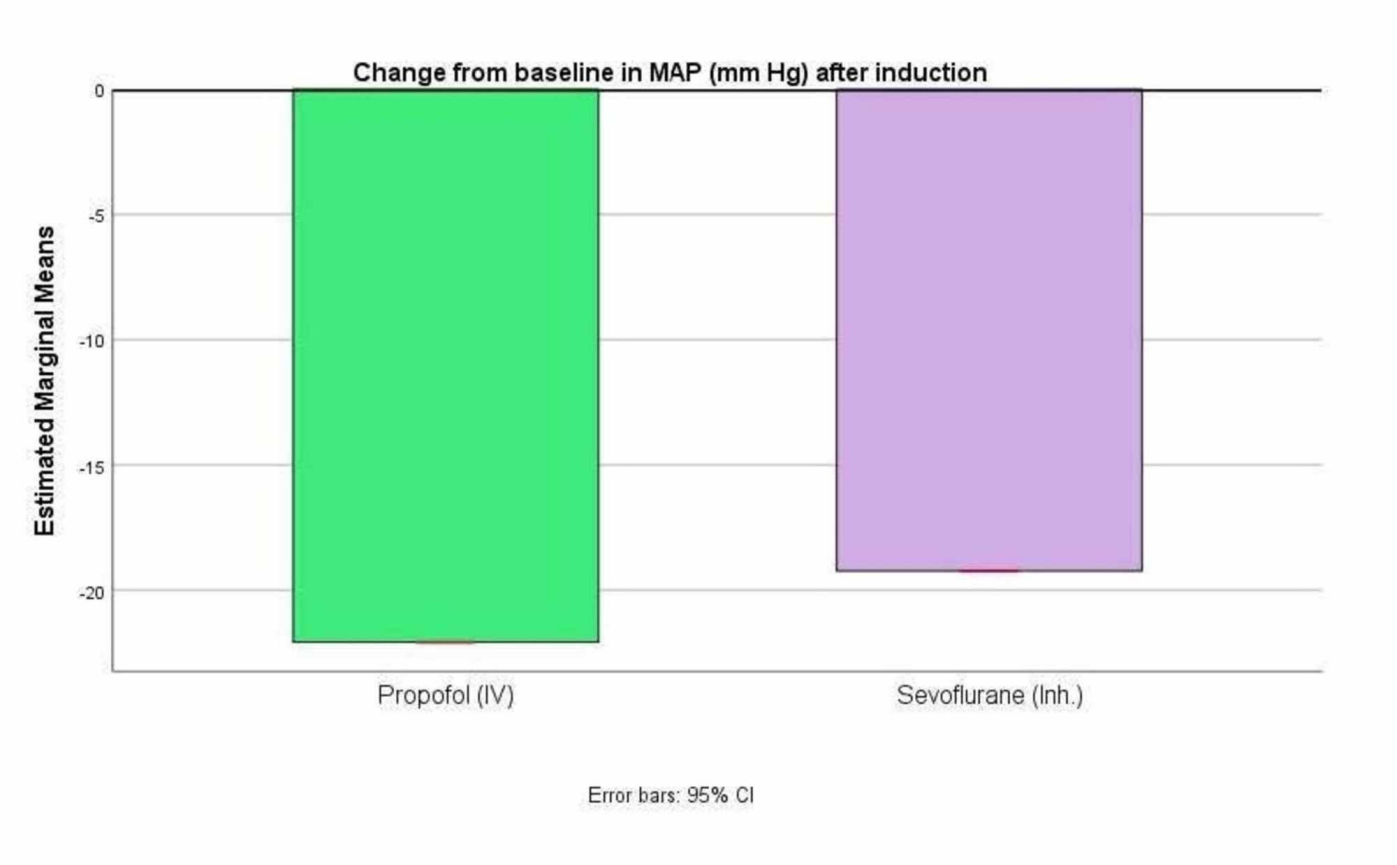
Percentage change in MAP from baseline after induction MAP - mean arterial pressure

Figure 6 shows the percentage change in MAP from baseline after induction. There was a greater reduction in MAP after induction with propofol compared to sevoflurane. However, the differences were not significant (p>0.05).

Table 2 shows the estimated means for the hemodynamic parameters for pooled data at different time points. The pooled mean estimates of MAP with sevoflurane was 82.52 and with propofol was 79.14 (p=0.028, significant) which suggests a greater stabilization of MAP with sevoflurane. However, the effect size was very small (0.063), which probably could be due to the inadequate power of the study. The pooled mean estimates for HR were similar in the two groups (p=0.173).

**Table 2:** Estimated means for hemodynamic parameters (pooled) * Repeat measures analysis of covariance (ANCOVA) with covariates evaluated at the following values: Age (years) = 33.88, Gender = 1.39, Body weight = 60.03. IV - intravenous; IH - inhalational; ANCOVA - analysis of covariance; CI - confidence interval

	Inducing agent	Mean	SE	95% CI for mean	Mean difference	p*	Effect size	Power
Heart rate (per min.)	Propofol (IV)	76.04	1.75	(72.54 - 79.53)	3.451	0.173	0.025	0.274
	Sevoflurane (IH)	72.59	1.75	(69.09 - 76.08)				
Systolic blood pressure (mm Hg)	Propofol (IV)	105.93	1.55	(102.83 - 109.02)	-8.059	0.001	0.150	0.948
	Sevoflurane (IH)	113.99	1.55	(110.89 - 117.08)				
Diastolic blood pressure (mm Hg)	Propofol (IV)	65.75	1.02	(63.73 - 67.78)	-1.030	0.480	0.007	0.108
	Sevoflurane (IH)	66.78	1.02	(64.76 - 68.81)				
Mean arterial pressure (mm Hg)	Propofol (IV)	79.14	1.05	(77.04 - 81.25)	-3.373	0.028	0.063	0.599
	Sevoflurane (IH)	82.52	1.05	(80.42 - 84.62)				

Table 3 shows the descriptives for percent change in hemodynamic parameters from baseline to post-induction in the two groups. There was a reduction in heart rate with sevoflurane by 9.71% after induction as against propofol, which had no effect on HR after induction. There were greater but not significant reductions (p>0.05) in SBP, DBP, and MAP with propofol as compared to sevoflurane.

**Table 3:** Percent change in hemodynamic parameters from baseline to post-induction IV - intravenous; IH - inhalational; SD - standard deviation; ANOVA - analysis of variance

	IV propofol (n=40)		IH sevoflurane (n=40)		ANOVA	
	Mean	SD	Mean	SD	F	p
Heart rate (per min.)	-0.70	15.89	-9.71	13.05	7.647	0.007
Systolic blood pressure (mm Hg)	-21.87	8.60	-20.93	9.09	0.222	0.639
Diastolic blood pressure (mm Hg)	-25.16	19.59	-16.76	19.17	3.759	0.056
Mean arterial pressure (mm Hg)	-23.93	12.90	-19.23	10.08	3.293	0.073

Table 4 shows the response for acceptance by patients in the two groups. Although sevoflurane was associated with unpleasant smell (5.0%) and feeling (12.5%), 10.0% of patients with propofol reported pain on injection (p=0.041, significant). The proportion of patients who reported the induction procedure unpleasant was similar (p=0.326, not significant) with sevoflurane (12.5%) and propofol (10.0%). A total of 36 (90.0%) patients reported they would prefer propofol for a repeat procedure, whereas with sevoflurane 34 (85.0%) patients reported the same (p=0.202, not significant).

**Table 4:** Acceptance by patients in the two groups IV - intravenous; IH - inhalational

	IV propofol (n=40)		IH sevoflurane (n=40)		Chi-square test	
	No.	%	No.	%	Chi-square	p
Opinion on induction procedure						
Mask unpleasant	0	-	5	12.5%	5.267	0.022
Smell unpleasant	0	-	2	5.0%	2.026	0.157
Pain on injection	4	10.0%	0	-	4.158	0.041
Opinion on anesthetic						
Pleasant	36	90.0%	33	82.5%	2.242	0.326
Indifferent	0	-	2	5.0%		
Unpleasant	4	10.0%	5	12.5%		
Choice of anesthetic for similar procedure in the future						
Same	36	90.0%	34	85.0%	3.200	0.202
Different	0	-	3	7.5%		
No preference	4	10.0%	3	7.5%		

**Figure 7:**
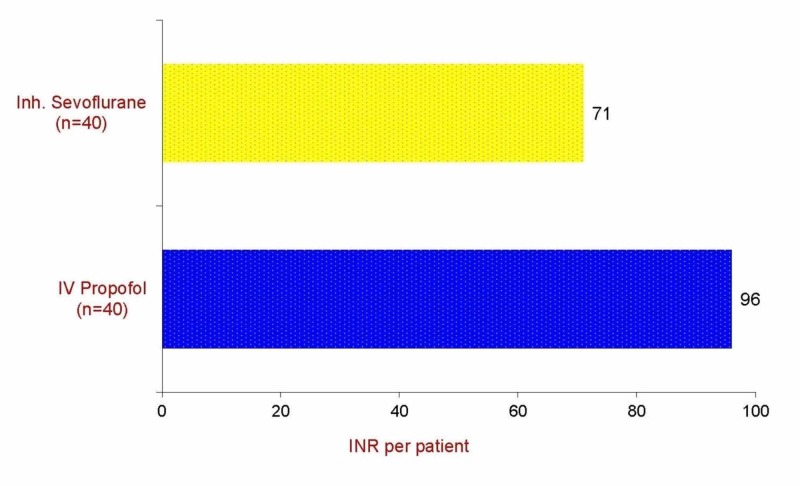
Mean cost (INR) for induction of anesthesia INR - Indian rupee; IV - intravenous; Inh. - inhalational

Figure 7 shows the mean cost (INR) for the induction of anesthesia. The per-unit cost of propofol is higher than that for sevoflurane when used for induction of anesthesia.

## Discussion

The choice of anesthetic could be different for patients with different ages and their underlying conditions [9]. Hemodynamic changes occurring during surgical anesthesia is a great cause of concern for both surgeons and anesthetists since it may be associated with various complications [10]. Techniques and methods used in the induction of anesthesia are designed to minimize hemodynamic instability, especially in geriatric patients [11].

Sevoflurane is an inhalational anesthetic drug having the least undesirable effects on hemodynamic changes and could be a better alternative to IV propofol, especially in patients prone to cardiovascular derangement. Propofol is a widely accepted induction agent and is favored for its early and "clear-headed" recovery facilitating early discharge. However, its potent depressing effects on the cardiovascular system, especially in combination with opioids, are little. Hence, due to its ability to reduce mean arterial pressure, propofol is being used along with ketamine or etomidate to reduce its effect on hemodynamic instability [12]. With the introduction of inhalational sevoflurane in anesthesia practice, a new era in the induction of anesthesia has begun due to its ability to smooth induction and early awakening [8, 13]. Although studies have demonstrated a favorable profile of sevoflurane over propofol as an inducing, maintenance, and recovery agent for outpatient anesthesia, there are limited studies on its effect on hemodynamic stability [14]. In this prospective, randomized comparative clinical study, we compared the effects of inhalational sevoflurane and IV propofol used for the induction of general anesthesia in adult patients. We observed that there was a significant decrease in the SBP, DBP, and MAP with propofol and not with sevoflurane after induction and up to five minutes after intubation. Although heart rate was increased with propofol and decreased marginally with sevoflurane, it may not be detrimental to cardiovascular stability. However, we observed that with sevoflurane, intubation led to a greater rise in heart rate, which continued for one minute and then slowly came down. With propofol, the raised heart rate after intubation almost immediately started falling but remained higher than the sevoflurane group at each corresponding time intervals. This pattern with propofol could be due to the resetting of baroreceptors, inhibition of sympathetic activity, and increased venous capacities. The rise in MAP due to intubation was similar in both groups, whereas, at five minutes after intubation, the fall in MAP with propofol was greater compared to sevoflurane (p<0.05). These results are indicative of better hemodynamic stability after induction with inhalational sevoflurane. Sevoflurane has attributes that facilitate rapid, smooth inhaled induction, has low blood gas solubility, the relative absence of pungency, and a vaporizer with high overpressure capability [15, 16]. The non-pungent odor of sevoflurane improves its acceptance for inhalation for most patients [17]. Our results suggest that sevoflurane is hemodynamically more stable and cost-effective than propofol and can be used as an induction technique in adult patients. Frink et al. also have drawn similar conclusions in their comparative study [18].

Patient acceptance is an important criterion for the selection of an inducing agent. We assessed the acceptance of anesthesia induction from the patient’s perspective and found it comparable in the two groups. About 90% of patients in the propofol group and 85% in sevoflurane groups were willing to receive the same anesthetic in the future. These findings agree with those by Sloan et al., who reported that sevoflurane odor to be pleasant, and it was popular among almost all patients [19]. However, Thaiwaiteset al. reported that patients described the smell of sevoflurane as unpleasant [8]. The introduction of newer inhalational agents has led to several pharmacoeconomic comparative studies to determine the relative cost. Factors that must be considered when studying the cost of these new volatile agents include high acquisition costs, although the use of a low fresh gas flow circle system may reduce the amount of this agent used. This, in turn, may have cost implications in those situations where rapid recovery has financial implications. To date, the available pharmacoeconomic data concerning propofol in day-case surgery consider only acquisition cost or per-minute administration costs of the drug. However, in considering only drug acquisition costs, these studies fail to evaluate other costs and benefits involved in anesthesia; various costs that should be considered for a complete economic evaluation of propofol include equipment, the cost of treating drug-related adverse effects, and staff salaries. The whole issue of whether anesthetic agents with rapid recovery profiles actually do decrease costs through an effect on recovery room stay is unclear. Similarly, Eger et al. reported that desflurane might be used in low flow circle systems with cost advantages, but it may be undesirable in the case of sevoflurane, due to its reaction with soda lime [20]. The cost of an inhaled anesthetic is not as simple to determine as that of an intravenous drug. The amount of inhaled anesthetic used can be calculated based on the anesthetic drug concentration and fresh gas flow, and this method has been used previously to assess the cost of induction of sevoflurane anesthesia [8]. In practice, the cost of intravenous induction depends on how much induction agent is actually drawn up and whether the remaining ampoule is discarded. We calculated the cost of propofol on the basis of the exact amount (ml) of propofol required. We observed that the induction of anesthesia with sevoflurane was significantly less costly compared with propofol.

The findings of our study are limited in terms of its generalizability due to the small sample size. The effect size and the power achieved with the analysis for the primary outcome were very limited, and further studies are warranted.

## Conclusions

Sevoflurane seems to have a better hemodynamic stability after induction compared to IV propofol. The patient acceptance is similar with the two inducing agents, but inhalational sevoflurane is more cost effective than IV propofol. However, further studies with large sample are required to substantiate these effects.
